# Bcl-2 inhibition sensitizes triple-negative human breast cancer cells to doxorubicin

**DOI:** 10.18632/oncotarget.25370

**Published:** 2018-05-22

**Authors:** Touko Inao, Yuichi Iida, Tamami Moritani, Tamio Okimoto, Ryosuke Tanino, Hitoshi Kotani, Mamoru Harada

**Affiliations:** ^1^ Department of Immunology, Shimane University Faculty of Medicine, Shimane, Japan; ^2^ Department of Breast Surgery, Takasago City Hospital, Hyogo, Japan; ^3^ Division of Medical Oncology & Respiratory Medicine, Department of Internal Medicine, Shimane University Faculty of Medicine, Shimane, Japan

**Keywords:** breast cancer, triple-negative, Bcl-2, ABT-199, doxorubicin

## Abstract

Breast cancers can be divided into several types. Because triple-negative breast cancer (TNBC) is the most refractory to current anti-cancer therapies, efficient treatment has been urgently required. Members of the Bcl-2 family play pro- and anti-apoptotic roles in mitochondria-mediated apoptosis. Some Bcl-2 family members are expressed in breast cancer and influence the response to anti-cancer therapies. In this study, we investigated whether Bcl-2 inhibition could sensitize TNBC cells to the genotoxic drug doxorubicin (DR). Treatment with a combination of the Bcl-2 inhibitor ABT-199 and DR synergistically decreased the viability of the TNBC cell lines MDA-MB-231 and BT-549. In an apoptosis assay, the combination treatment resulted in only a marginal effect in BT-549 cells, whereas drastic apoptosis was induced in MDA-MB-231 cells treated with both ABT-199 and DR. Both caspase-8 and -9 were involved in the combination treatment-induced apoptosis. Short interfering RNA-mediated knockdown of Bcl-2 increased the sensitivity of both cell lines to DR. The combination treatment also significantly decreased the colony-forming ability of the TNBC cell lines. In a xenograft mouse model, oral administration of ABT-199 augmented the DR-induced antitumor effect on subcutaneously established MDA-MB-231 cells. These results indicate that the combination of DR with Bcl-2 inhibitors, including ABT-199, may be a promising treatment modality for TNBC patients.

## INTRODUCTION

Breast cancer is the most common cancer among women worldwide and can be divided into several groups based on molecular analyses. One type is triple-negative breast cancer (TNBC), which comprises 15–20% of all breast cancers [1]. TNBC cells lack expression of the estrogen receptor (ER), progesterone receptor, and human epidermal growth factor receptor-2. Due to a lack of effective therapy [1, 2], progression-free and overall survival rates of TNBC patients are poor [3]. Therefore, a novel effective treatment modality against TNBC is urgently required.

Apoptosis is primarily induced in cancer cells through two major pathways: extrinsic and intrinsic pathways [4]. Fas ligand (FasL) and tumor necrosis factor-related apoptosis-inducing ligand (TRAIL) provide a death signal via the extrinsic apoptotic pathway, activating caspase-8 in cancer cells. In contrast, cytotoxic drugs and high-dose radiation damage DNA and mitochondria, resulting in activation of the intrinsic caspase-9-mediated apoptotic pathway. Although several molecules participate in mitochondria-mediated apoptosis [5–7], Bcl-2 family members play critical roles in this type of apoptosis [8, 9]. The family of Bcl-2-related anti-apoptotic proteins, which includes Bcl-2, Bcl-X_L_, Bcl-w, and Mcl-1, has been shown to contribute to the chemotherapy resistance of several cancer cells [9]. To target members of the Bcl-2 family, several small molecule inhibitors have been developed. ABT-737 and ABT-263 (navitoclax) are small molecule inhibitors of Bcl-2, Bcl-X_L_, and Bcl-w. Several reports have demonstrated the efficacy of these inhibitors against both hematological malignancies and several types of solid tumors [10–17]. Regardless of the efficacy of these agents, thrombocytopenia due to Bcl-X_L_ inhibition was the major dose-limiting toxicity and was dose-related [15, 18]. Alternatively, ABT-199 (venetoclax) is a Bcl-2 inhibitor that spares platelets [19]. ABT-199 is currently one of the most promising agents in clinical development for hematological malignancies [10]. Interestingly, Bcl-2 is commonly associated with ER-positive breast tumors [20] and has been recognized as a favorable prognostic marker in breast cancer [21, 22]. In addition, the expression of Bcl-2 predicts the efficacy of adjuvant chemotherapy in breast cancer patients [23]. Furthermore, downregulation of Bcl-2 enhances the effects of chemotherapeutic agents in human breast cancer cells [24]. However, its potential role in TNBC has not yet been fully determined. Given that Bcl-2 is an anti-apoptotic protein, Bcl-2 could be a target molecule in the treatment of TNBC patients.

Current chemotherapy treatment for breast cancer consists of anthracycline and cyclophosphamide in combination with taxanes [25]. Because doxorubicin (DR), an anthracycline agent, has frequently been used in the treatment of breast cancer, it is therapeutically important to search for modalities that can enhance DR-induced antitumor effects in TNBC. In addition, patients with Bcl-2-negative TNBC were suggested to benefit from chemotherapy, including DR [26]. To this end, in this study, we determined whether the Bcl-2 inhibitor ABT-199 could augment the DR-induced antitumor effects in two TNBC cell lines, MDA-MB-231 and BT-549. Our results indicate that ABT-199 can enhance DR-induced antitumor effects: induction of apoptosis and inhibition of the colony-forming ability of TNBC cells. Furthermore, ABT-199 enhanced DR-induced growth suppression of MDA-MB-231 cells in a xenograft mouse model. These results reveal that Bcl-2 inhibition can potentiate the antitumor effects of DR in TNBC.

## RESULTS

### Combination treatment with DR and ABT-199 decreased the viability of TNBC cells

**Figure 1:**
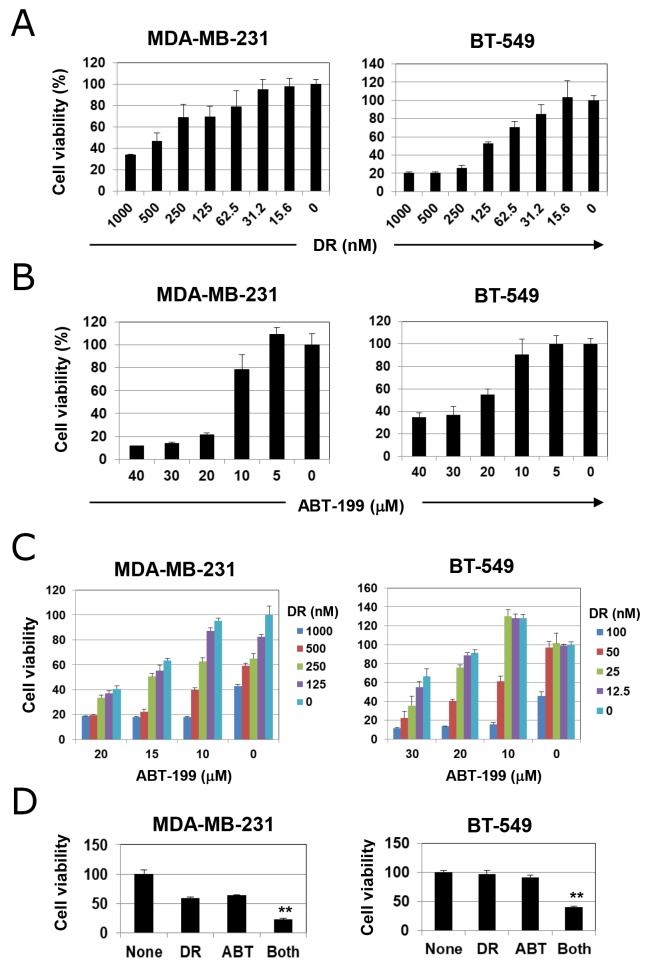
Cell viability of triple-negative breast cancer (TNBC) cells treated with doxorubicin (DR) and/or ABT-199 **(A)** TNBC cells were cultured with the indicated doses of DR (nM) for 48 h. **(B)** TNBC cells were cultured with the indicated doses of ABT-199 (μM) for 48 h. **(C)** TNBC cells were cultured with the indicated doses of DR (nM) and/or ABT-199 (μM) for 48 h. In these experiments, cell viability (%) was determined using the WST-8 assay. The results are shown as the means ± standard deviation (SD) of three wells. Similar results were obtained in four separate experiments. **(D)** Representative results (MDA-MB-231 cells, 500 nM DR and 15 μM ABT-199; and BT-549 cells, 50 nM DR and 20 μM ABT-199) are shown. ^**^p < 0.01.

We first examined the effects of DR and ABT-199 in two TNBC cell lines. DR decreased cell viability in a dose-dependent manner (Figure 1A). BT-549 cells were more sensitive to DR than MDA-MB-231 cells, whereas MDA-MB-231 cells were more sensitive to ABT-199 than BT-549 cells (Figure 1B). When cells were treated with both DR and ABT-199, viability was decreased even further compared with either treatment alone (Figure 1C). Representative results are shown in Figure 1D (*p*<0.01; control group versus the other groups). These results indicate that the combination of DR and ABT-199 can effectively decrease the viability of TNBC cells *in vitro*.

### Combination treatment with DR and ABT-199 triggered caspase-dependent apoptosis in TNBC cells

**Figure 2:**
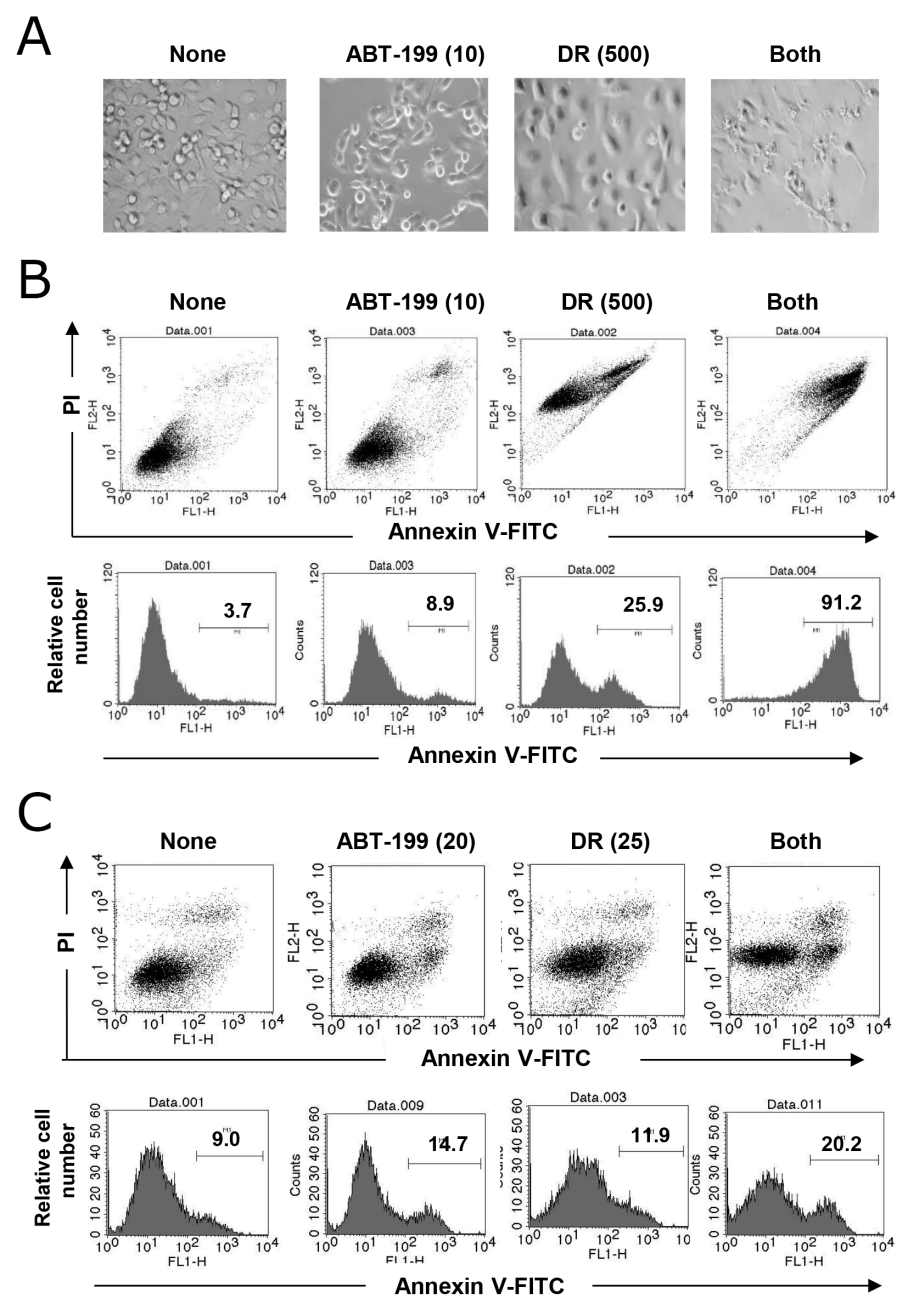
Apoptosis in TNBC cells following combination treatment with DR and ABT-199 **(A)** MDA-MB-231 cells were treated with 10 μM ABT-199 and/or 500 nM DR. After 2 days, images were obtained. **(B)** MDA-MB-231 cells were treated with 10 μM ABT-199 and/or 500 nM DR. After 2 days, cells were stained with fluorescein isothiocyanate (FITC)-conjugated annexin V and propidium iodide (PI), and flow cytometry was performed. **(C)** Similarly, BT-549 cells were treated with 20 μM ABT-199 and/or 25 nM DR, and cells were analyzed by flow cytometry. The numbers represent the percentages of annexin V-positive cells.

Microscopic observation of MDA-MB-231 cells revealed that DR treatment increased cell size and treatment with both DR and ABT-199 resulted in a drastic destruction of MDA-MB-231 cells (Figure 2A). Therefore, we performed an apoptosis assay following annexin V-fluorescein isothiocyanate (FITC) and propidium iodide (PI) staining. As shown in Figure 2B, although treatment with DR or ABT-199 alone slightly increased the percentage of annexin V-positive MDA-MB-231 cells, combination treatment with DR and ABT-199 drastically increased the percentage of annexin V-positive cells. Unexpectedly, DR treatment increased the longitudinal FL2 intensity of MDA-MB-231 cells likely because PI emitted red fluorescence. Combination treatment with DR and ABT-199 increased the percentage of annexin V-positive BT-549 cells, whereas the induction of apoptosis was not as marked in MDA-MB-231 cells (Figure 2C). PI staining slightly increased the FL2 intensity.

**Figure 3:**
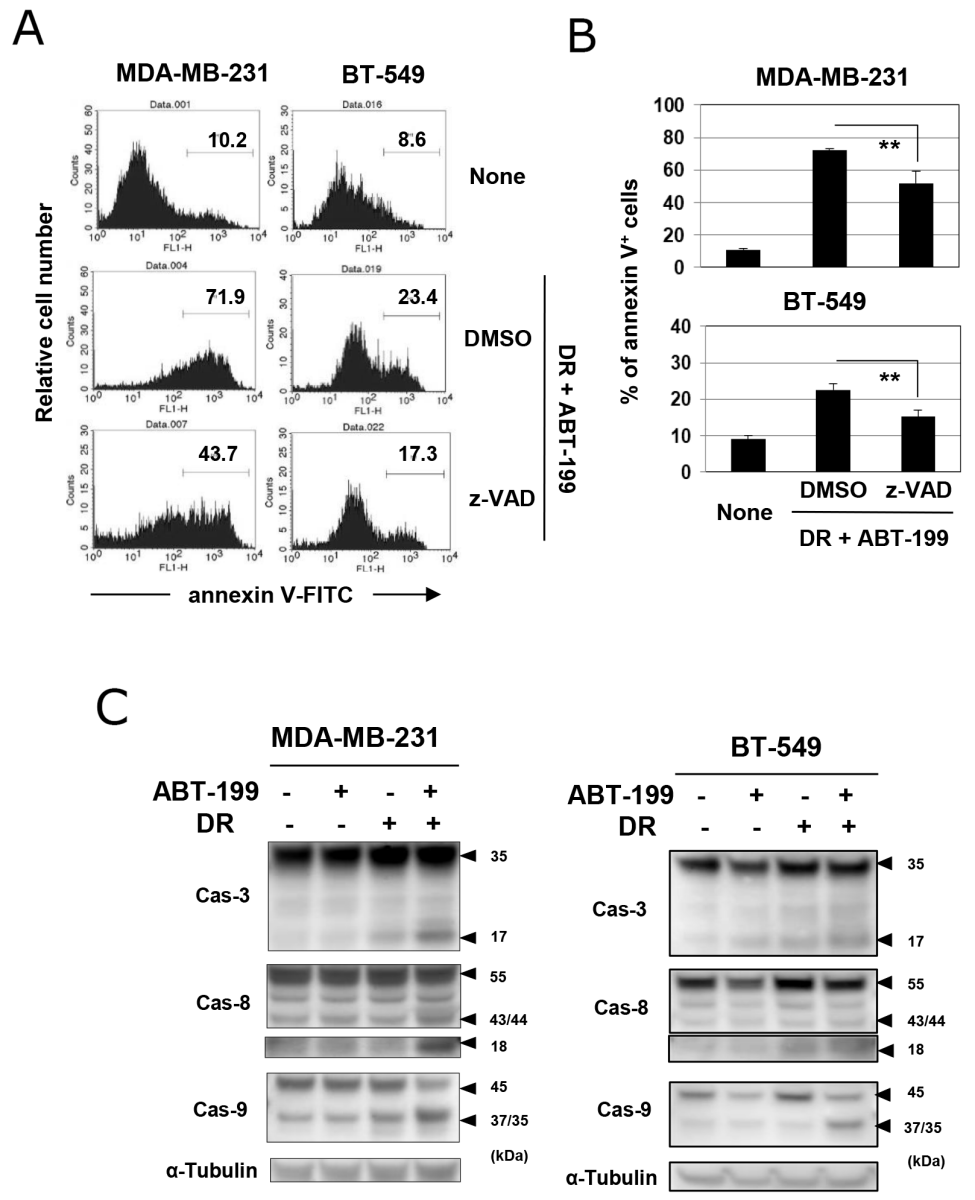
Caspase-dependent apoptosis in TNBC cells treated with DR and ABT-199 **(A)** TNBC cells were treated with both DR and ABT-199 for 48 h (MDA-MB-231 cells, 500 nM DR and 10 μM ABT-199; and BT-549 cells, 25 nM DR and 20 μM ABT-199). In some groups, 20 μM z-VAD or dimethyl sulfoxide (DMSO), as a vehicle control, was added. After staining with FITC-conjugated annexin V/PI, the cells were analyzed by flow cytometry. The numbers represent the percentages of annexin V-positive cells. **(B)** The results are shown as the means ± SD of three wells. ^**^p < 0.01. **(C)** TNBC cells were treated with ABT-199 and/or DR at the doses described above. After 24 h, cells were harvested and examined for their expression of caspase-3, -8, and -9 by immunoblotting. α-Tubulin was used as a control.

We next determined whether cell death induced by the combination of DR and ABT-199 was due to caspase-dependent apoptosis. Although the inhibitory efficacy was only partial, the addition of the pan-caspase inhibitor, z-VAD, decreased the percentages of annexin V-positive cells in both cell lines treated with DR and ABT-199 (Figure 3A and 3B) (*p*<0.01; z-VAD versus DMSO). Additionally, immunoblotting revealed that the combination treatment increased the cleavage of caspase-3, -8, and -9 in both cell lines (Figure 3C), indicating that the combination of DR and ABT-199 induces caspase-dependent apoptosis of TNBC cells, at least partially.

### Genetic knockdown of Bcl-2 increased the sensitivity of TNBC cells to DR

**Figure 4:**
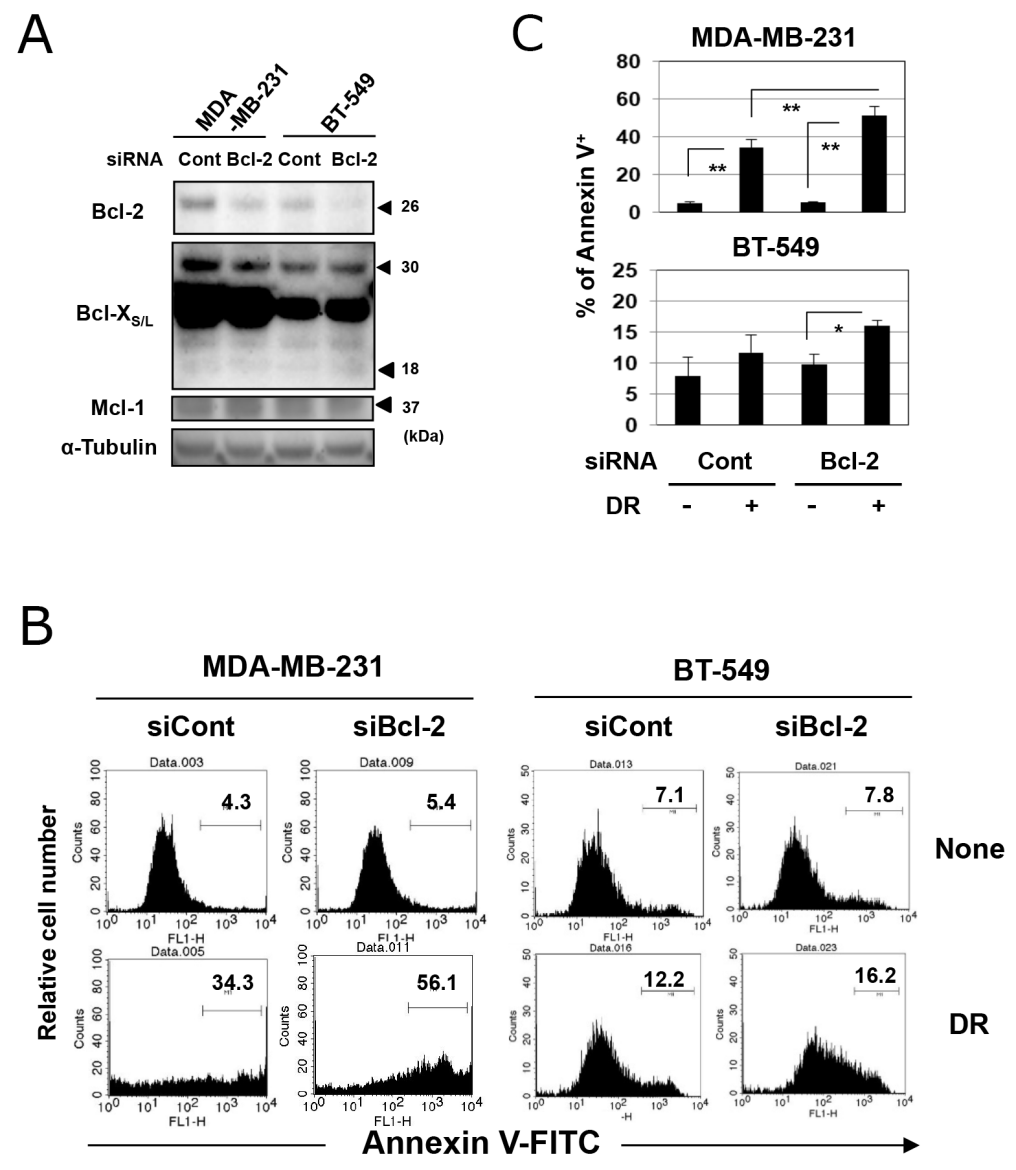
Genetic knockdown of Bcl-2 increased the sensitivity of TNBC cells to DR **(A)** TNBC cells were transfected with Bcl-2 short interfering RNA (siRNA) or control siRNA. After 2 days, cells were harvested and examined for expression of Bcl-2, Bcl-X, and Mcl-1. α-Tubulin was used as a control. **(B)** Two days after siRNA transfection, MDA-MB-231 and BT-549 cells were cultured with 500 and 25 nM DR, respectively, for 48 h. Following staining with FITC-conjugated annexin V/PI, flow cytometry was performed. The numbers represent the percentages of annexin V-positive cells. **(C)** The results are shown as the means ± SD of three wells. ^*^p < 0.05, ^**^p < 0.01. NS, not significant.

The treatment with ABT-199 showed no effect on the protein expression of Bcl-2 in two TNBC cell lines *in vitro* (Supplementary Figure 1). Therefore, we next determined whether genetic knockdown of Bcl-2 could sensitize TNBC cells to DR. Both cell lines were positive for Bcl-2 at the protein level, and transfection with Bcl-2-targeted short interfering RNA (siRNA) decreased the expression of Bcl-2 (Figure 4A). Knockdown of Bcl-2 showed no definite effect on the expression of Bcl-X_S/L_ and Mcl-1. Bcl-2 knockdown in MDA-MB-231 cells significantly increased the percentage of annexin V-positive DR-treated cells compared with control siRNA-transfected and DR-treated cells (Figure 4B and 4C) (*p*<0.01). Although no difference in the percentage of annexin V-positive cells was observed between control siRNA-transfected untreated BT-549 cells and control siRNA-transfected DR-treated BT-549 cells, Bcl-2 knockdown significantly increased the sensitivity of BT-549 cells to DR (*p*<0.05).

### Combination treatment with DR and ABT-199 decreased the colony-forming ability of TNBC cells

**Figure 5:**
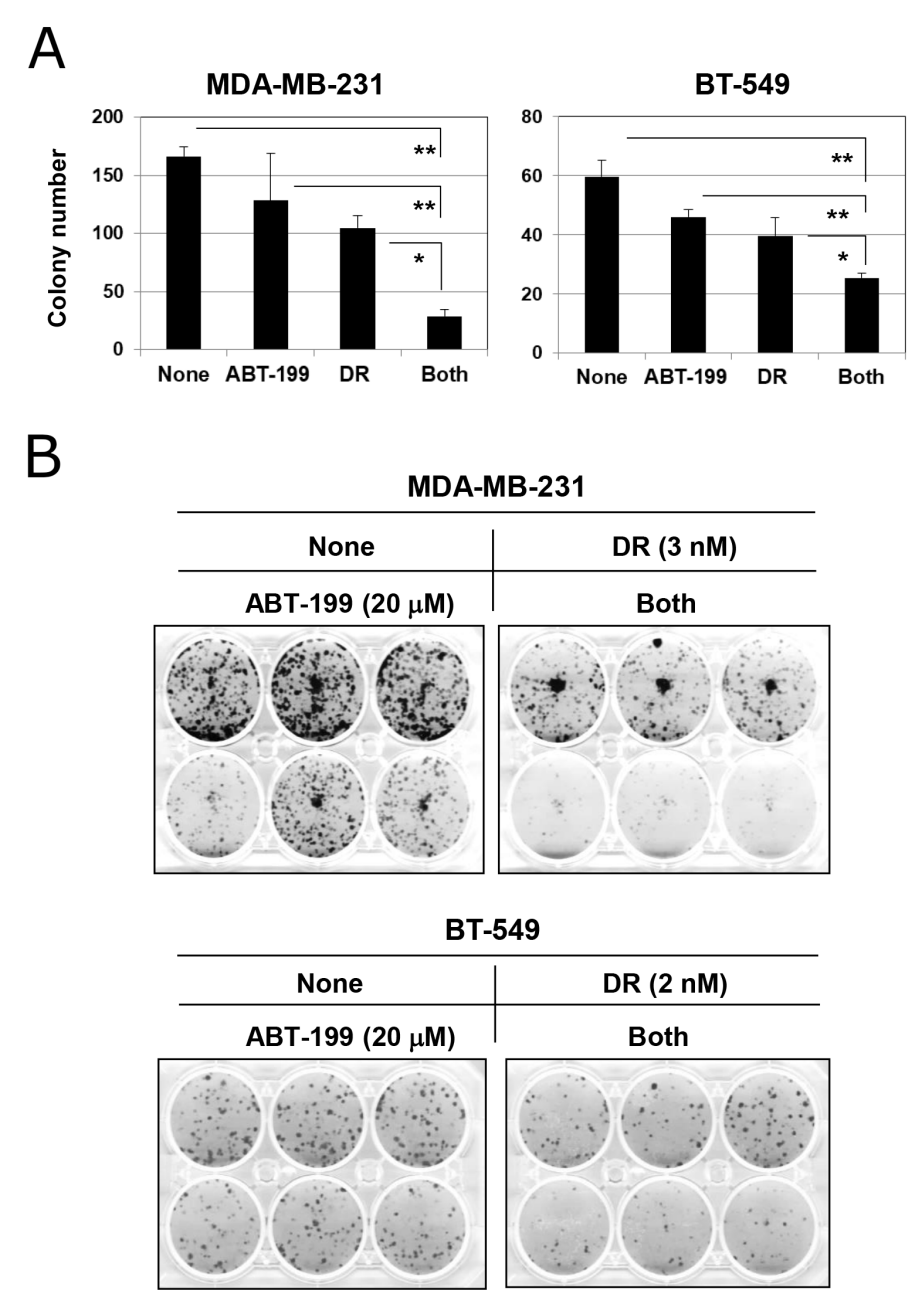
Combination treatment with DR and ABT-199 impaired the colony-forming ability of TNBC cells **(A)** TNBC cells were cultured with the indicated doses of DR and/or ABT-199 (MDA-MB-231 cells, 3 nM DR and 20 μM ABT-199; and BT-549 cells, 2 nM DR and 20 μM ABT-199). After 2 days, the supernatant was replaced with new culture medium without reagents and cultured for an additional 7 or 10 days. Colonies were counted after staining with 0.05% crystal violet. The results are shown as the means ± SD of three wells. ^*^p < 0.05, ^**^p < 0.01. **(B)** Representative results are shown.

We next examined the effect of combination treatment with DR and ABT-199 on the colony-forming ability of TNBC cells. Although higher doses of DR (500 and 25 nM for MDA-MB-231 and BT-549 cells, respectively) were used in the apoptosis assay (Figure 2), 2-day treatment at such doses drastically abolished the colony-forming ability (data not shown). Therefore, based on the results of dose titration experiments, MDA-MB-231 and BT-549 cells were treated with 3 and 2 nM DR, respectively. Although treatment with DR alone decreased colony numbers in both cell lines to some degree, the combination treatment significantly suppressed the colony-forming ability of both cell lines more profoundly (Figure 5A) (*p*<0.01; combination group versus ABT-199 group and *p*<0.05; combination group versus the DR group). Representative results are shown in Figure 5B. This suppression was more apparent in MDA-MB-231 cells compared with BT-549 cells. These results indicate that combination treatment with DR and ABT-199 can exert antitumor effects on TNBC cells via inhibition of their colony-forming ability.

### Combination therapy with DR and ABT-199 suppressed the tumor growth of MDA-MB-231 cells *in vivo*

**Figure 6:**
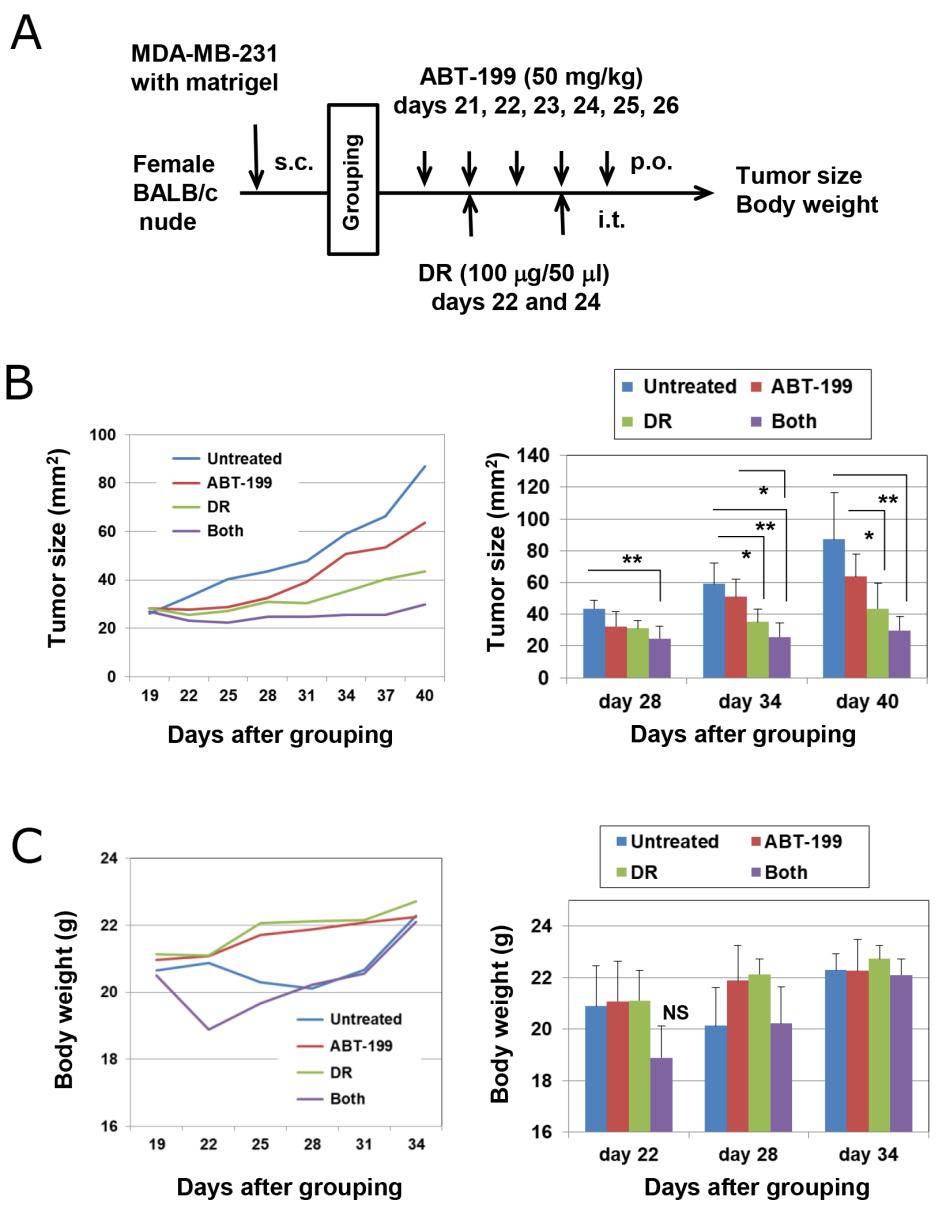
Combination treatment with DR and ABT-199 suppressed the growth of MDA-MB-231 cells *in vivo* **(A)** Experimental protocol. **(B)** (Left) Tumor growth of MDA-MB-231 cells is shown. Each group consists of six BALB *nu/nu* mice. (Right) Tumor size, product of two perpendicular diameters, was measured every 3 or 4 days. The results are shown as the means ± SD of six mice. ^*^p < 0.05, ^**^p < 0.01 (ANOVA). **(C)** Body weight was measured every 3 or 4 days. The results are shown as the means ± SD of six mice. NS, not significant.

Finally, we determined whether combination treatment with DR and ABT-199 could exert an antitumor effect against subcutaneously established MDA-MB-231 cells using a xenograft mouse model. Nude mice were subcutaneously inoculated with MDA-MB-231 cells into the right flank and divided into four groups on day 20. ABT-199 (50 mg/kg) was administered orally on days 21, 22, 23, 24, 25, and 26. DR (100 μg/50 μl) was injected intratumorally on days 22 and 24 (Figure 6A). Although treatment with ABT-199 alone failed to show tumor suppression and the injection of DR alone suppressed tumor growth on days 34 and 40 compared with the untreated control (*p* < 0.05), the combination of DR and ABT-199 significantly suppressed tumor growth (p < 0.01 versus control group) (Figure 6B). In terms of body weight, although the combination therapy transiently decreased body weight, it recovered soon after (Figure 6C). These results indicate that combination therapy with DR and ABT-199 can inhibit the tumor growth of human TNBC cells *in vivo*.

## DISCUSSION

In this study, we investigated the therapeutic effect of combining the genotoxic drug DR and the Bcl-2 inhibitor ABT-199 in two human TNBC cell lines and revealed that ABT-199 could effectively enhance the susceptibility of TNBC cells to DR.

DR is a frequently used cytotoxic drug for the treatment of breast cancer. However, since TNBC is generally refractory to current anti-cancer therapies, including DR, an efficient treatment modality has been urgently required. Bcl-2 family members play multiple roles in apoptosis. ABT-199 is a Bcl-2 specific inhibitor that can be administered orally [18]. Other Bcl-2 family inhibitors, such as ABT-737 and ABT-263, inhibit not only Bcl-2 but also Bcl-X_L_ and Bcl-w [27]. We previously reported that ABT-737 and ABT-263 could sensitize human prostate cancer cells and pancreatic cancer cells to docetaxel and TRAIL, respectively [28, 29]. However, Bcl-X_L_ is important in the survival of platelets, and a gradual reduction of Bcl-X_L_ results in apoptosis of senescent platelets [30]. Therefore, in a clinical trial of ABT-263 (navitoclax), it was shown that doses of ABT-263 must be reduced to avoid severe thrombocytopenia [15, 17]. In contrast, in a phase II study of ABT-199 monotherapy for patients with acute myelogenous leukemia, common adverse events did not include severe thrombocytopenia [31]. Based on these lines of evidence, we tested the ability of the Bcl-2 inhibitor ABT-199 to augment the antitumor effects of DR on TNBC.

Cytotoxic chemotherapeutic drugs generally induce mitochondria-mediated apoptosis. Overexpression of Bcl-2 proteins can induce drug resistance in many types of epithelial cancer cells because Bcl-2 decreases mitochondrial apoptosis by stabilizing mitochondrial permeability [32]. Bcl-2 proteins inhibit the release of cytochrome c from mitochondria into the cytosol to activate caspase-3 and -9. In this study, we showed that combination treatment with DR and ABT-199 induced caspase-dependent apoptosis in TNBC cells. Using immunoblotting, we showed that this combination treatment induced cleavage of both caspase-8 and -9 (Figure 3C). In general, cytotoxic drugs, including DR, damage DNA and mitochondria, resulting in activation of intrinsic caspase-9-mediated apoptosis. Bcl-2 family members play a critical role in this type of apoptosis [8, 9], whereas the extrinsic apoptotic pathway, which can be induced by FasL and TRAIL, activates caspase-8. Interestingly, drug-induced endoplasmic reticulum stress can induce apoptosis in cancer cells via death receptor-mediated activation of caspase-8 [33]. In reverse, activation of caspase-8 can trigger intrinsic caspase-9-mediated apoptosis through truncation of Bid [34, 35]. In general, the induction of apoptosis can involve both pathways.

Mutation or loss of the *p53* gene is frequently observed in many types of human cancers and is associated with poor prognosis and chemoresistance [36, 37]. Chemotherapy-induced killing of cancer cells requires activation of BH3-only proteins, such as PUMA and NOXA, by upstream signaling mediators including p53 [38]. These upstream signal activators are frequently mutated, lost, or silenced during tumor development or during emergence of therapy-resistant cancer cells [38]. To overcome such resistance, a new class of therapeutics, known as BH3-mimetics such as ABT-199, has been developed [38]. In this study, we used two human TNBC cell lines, MDA-MB-231 and BT-549, both of which carry a mutation in the *p53* gene [39]. Our results suggest that combination treatment with DR and ABT-199 is effective even against breast cancers with a mutant *p53* gene.

Although tumors expressing high levels of Bcl-2 respond effectively to the Bcl-2 family inhibitor ABT-737 in combination with docetaxel, tumors expressing low levels of Bcl-2 showed no response [40]. Additionally, ABT-737 monotherapy has been shown to be effective in treating some malignancies, including follicular lymphoma and chronic lymphocytic leukemia [27], whereas this inhibitor was not effective against breast tumors in xenograft models [40]. These data suggest that Bcl-2 inhibition is effective against lymphoid malignancies in which Bcl-2 is a key oncogenic driver [41], but that this inhibition alone is not as effective in the treatment of breast cancer. However, as shown in this study, Bcl-2 inhibition can effectively sensitize TNBC cells to the cytotoxic drug DR. Given that Bcl-2 is overexpressed in approximately 75% of breast cancers [22], this combination therapy appears promising.

It has been reported that targeting Bcl-2 with ABT-199 can increase therapeutic effects of tamoxifen on ER^+^ breast cancer in primary breast tumor xenograft models [14]. In addition, *in vivo* silencing of Bcl-2 with nanoliposomal Bcl-2 siRNA can inhibit the growth of xenografted ER^-^ and ER^+^ breast cancers [42]. In this study, combination treatment with DR and ABT-199 was effective in a xenograft model of triple-negative MDA-MB-231 cells, compared with either therapy alone (Figure 6). Although transient body weight loss was observed in mice following combination therapy, this adverse event was soon recovered. These lines of *in vivo* evidence suggest that Bcl-2 is a promising target in treating not only ER^+^ breast cancer but also TNBC.

Overall, we investigated the sensitizing effect of the Bcl-2 inhibitor ABT-199 on DR-induced antitumor effects in two TNBC cell lines. Our data indicate that ABT-199 effectively enhanced DR-induced anti-tumor effects both *in vitro* and *in vivo*. Given that the emergence of DR-resistance in patients with TNBC has been an important therapeutic hurdle, our findings provide a clue to overcome DR resistance. Finally, our results suggest that determining the expression of Bcl-2 may identify a subset of TNBC patients who may benefit from treatment with the Bcl-2 inhibitor ABT-199.

## MATERIALS AND METHODS

### Cell culture and reagents

Two human breast cancer cell lines (MDA-MB-231 and BT-549), which were kindly provided by Dr. K. Takenaga (Shimane University Faculty of Medicine), were maintained in DMEM medium (Sigma-Aldrich, St. Louis, MO) supplemented with 10% FCS (Invitrogen, Grand Island, NY) and 20 μg/ml gentamicin (Sigma-Aldrich) at 37°C in a humidified atmosphere with 5% CO_2_. ABT-199 was purchased from Chemie Tek (Indianapolis, IN, USA). DR was obtained from Sigma-Aldrich and diluted at first in dH2O and subsequently in PBS. The pan-caspase inhibitor z-VAD-FMK was purchased form Enzo Life Science (Farmingdale, NY, USA).

### Cell viability assay

Cell viability was measured using the 2-(2-methoxy-4 -nitrophenyl)-3-(4-nitrophenyl)-5-(2, 4-disulfophenyl)-2H-tetrazolium monosodium salt (WST-8) assay (Nacalai Tesque, Kyoto, Japan). Briefly, cells were seeded in flat-bottomed 96-well plates and doxorubicin and/or ABT-199 at the indicated doses was added. Two days later, 10 μl WST-8 solution was added to each well and the plates were incubated for an additional 3 h. The plates were read at a wavelength of 450 nm using a microplate reader (Beckman Counter, Brea, CA, USA).

### Photograph

Cancer cells were treated with ABT-199 and/or DR. After two days, photograph was taken using IX71, DP12, and TH4-100 (Olympus, Tokyo, Japan).

### Flow cytometric analysis

Apoptosis was measured using the annexin V-FITC Apoptosis Detection Kit (BioVision, Mountain View, CA) and propidium iodide (PI). Analysis was performed using a FACS Calibur flow cytometer (Becton Dickinson, Fullerton, CA). For inhibition assays, pan caspase inhibitor z-VAD-FMK (20 μM) was added at the initiation of culture. DMSO was used as a vehicle control.

### Immunoblot assay

Cells were lysed with the M-PER mammalian protein extraction reagent (Thermo Scientific, Rockford, IL) containing a protease inhibitor cocktail (Nacalai Tesque). Equal amounts of protein were resolved on 4–12% gradient or 12% SDS-PAGE gels, followed by transfer to polyvinylidene fluoride membranes. After blocking membranes, blots were incubated with the indicated primary antibodies: anti-Bcl-2 (sc-492; Santa cruz biotechnology [SCB], Dallas, Texas, USA, anti-Bcl-X_S/L_ (#633901; BioLegend, San Diego, CA, USA ), anti-Mcl-1 (sc-819; SCB ), anti-caspase-3 (9668; Cell Signaling Technology [CST], Danvers, MA, USA ), anti-caspase-8 (M032-3; Medical and Biological Laboratories, Nagoya, Japan), anti-caspase-9 (9508; CST), anti-β-actin (BioLegend ) or anti-α-tubulin (SCB). After washing, room temperature incubation of membranes for 30 min with either goat anti-rabbit or goat anti-mouse alkaline phosphatase-conjugated secondary antibodies (Invitrogen) were used to detect the primary antibodies. Protein bands were visualized using an ImageQuant LAS-4000 system (FujiFilm, Tokyo, Japan).

### Transfection of siRNA

Transfection of siRNA was performed using Lipofectamine™ RNAiMAX (Invitrogen, Grand Island, NY, USA) according to the manufacturer’s instructions. siRNAs targeting Bcl-2 were purchased from SCB and Invitrogen, respectively. Control siRNA (#6568) was purchased from CST. The transfected cells were used for the experiments 2 days after siRNA transfection.

### Colony-forming assay

Cells were seeded in flat-bottomed six-well plates with DR and/or ABT-199 at the indicated doses. Two days later, the medium was replaced with new medium without reagents, and the culture was continued for additional 9-12 days. Thereafter, colonies were fixated with methanol and stained with 0.05% crystal violet, then counted.

### *In vivo* xenograft model

BALB *nu/nu* female mice, purchased from CLEA Japan Inc. (Tokyo, Japan), were maintained under specific pathogen-free conditions. The protocol was approved by Committee on the Ethics of Animal Experiments of the Shimane University Faculty of Medicine (Permit Number: IZ26-107, IZ29-50). All efforts were made to minimize suffering. Mice were inoculated subcutaneously into the right flank with MDA-MB-231 (5×10^6^ cells) and Matrigel (Japan BD Biosciences, Tokyo, Japan) at a 1:1 volume ratio as a total volume of 100 μl. When the tumor diameter reached approximately 5 mm, the mice were pooled and divided into four groups on day 20. On the indicated days, these MDA-MB-231- bearing mice were treated with DR and/or ABT-199. As a vehicle control for DR, 100 μl PBS was injected intratumorally. As a vehicle control for ABT-199, 100 μl DMSO were orally administered using gavage. The tumor size and product of the two perpendicular diameters were measured twice weekly. Each group contained six mice.

### Statistical analysis

Data were statistically evaluated using unpaired two-tailed Student’s *t-*tests or an ANOVA together with Tukey’s test. A *p*-value less than 0.05 was considered to be statistically significant.

## SUPPLEMENTARY MATERIALS FIGURE


