# Supplementary material for: Bcl-2 inhibition sensitizes triple-negative human breast cancer cells to doxorubicin
Source: Oncotarget. 2018 May 22;9(39):25545–56. doi: 10.18632/oncotarget.25370 (PMC5986635; doi:10.18632/oncotarget.25370)
Supplement: Supplementary file 1 [file oncotarget-09-25545-s001.pdf]

Bcl-2 inhibition sensitizes triple-negative human breast cancer cells to doxorubicin

SUPPLEMENTARY MATERIALS

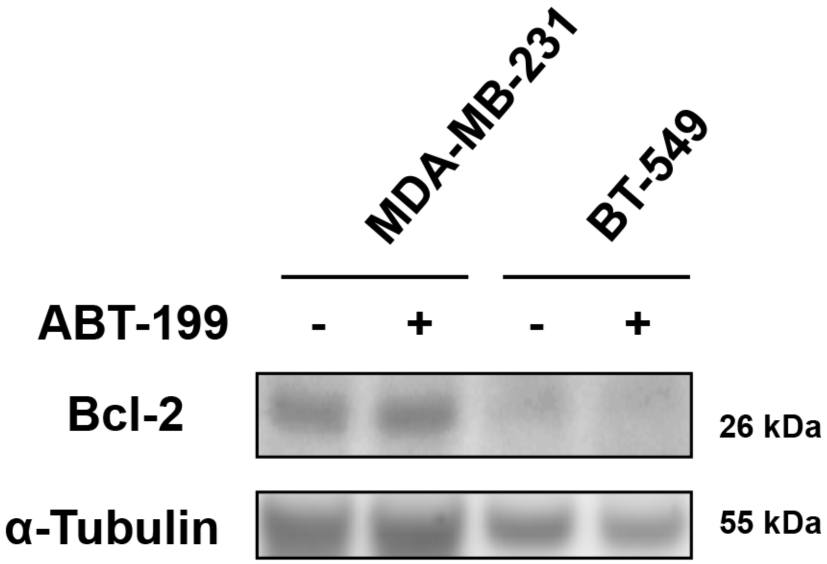

Supplementary Figure 1: No effect of ABT-199 on the protein expression of Bcl-2 in breast cancer cell lines. MDA-MB-231 and BT-459 cells were cultured with ABT-199 at the doses of 10 µM and 15 µM, respectively. After 48 h, the cells were harvested and examined for the protein expression of Bcl-2. α-Tubulin was used as a control.
